# Improved Multistage In-Motion Attitude Determination Alignment Method for Strapdown Inertial Navigation System

**DOI:** 10.3390/s19204568

**Published:** 2019-10-21

**Authors:** Haiyan Qiao, Meng Liu, Hao Meng, Mengjun Wang, Wei Ke

**Affiliations:** 1 College of Automation, Harbin Engineering University, Harbin 150001, China; qiaohaiyan623@163.com (H.Q.); menghao@hrbeu.edu.cn (H.M.); 2 Tianjin Navigation Instrument Research Institute, Tianjin 300131, China; 3 Hebei Hanguang Industry Co., Ltd., Handan 056000, China; hgzggsbgs@163.com (M.W.); kewei905@163.com (W.K.)

**Keywords:** initial alignment, In-Motion Attitude Determination Alignment (IMADA), dual velocity-modeling IMADA alignment, multistage alignment

## Abstract

This paper derives an improved multistage in-motion attitude determination alignment (IMADA) for strapdown inertial navigation system, which integrates the traditional IMADA and the designed dual velocity-modeling IMADA, as well as the multiple repeated alignment process, to address the principled model errors and the calculation errors of traditional $V^{b}$-aided IMADA. With the proposed algorithm, not only the designed drawbacks of traditional $V^{b}$-based IMADA can be solved, but also the degradation phenomenon of high-level alignment for multistage IMADA would be largely less. Moreover, the degradation of the alignment accuracy with the vehicle velocity is also removed. Finally, the 30 groups of car-mounted experiments and the Monte Carlo simulation experiments with the navigation-grade SINS are carried out to demonstrate the validity of the proposed algorithm. The results show that the number of the heading degradation of the second-level alignment is reduced to 10 as compared the traditional number 20. Moreover, the alignment accuracy of heading is improved by 23%. Even with the different speeds of 20 m/s, 60 m/s, 80 m/s, the heading alignment accuracies are 1.3063°, 1.3102°, 1.3564° and are still almost the same.

## 1. Introduction

Initial alignment to determine the initial attitude matrix between the body frame and navigation frame would be necessary for strapdown inertial navigation system (SINS). The alignment performance would directly affect the following navigation accuracy. Normally, this process is composed of two phases: coarse alignment and fine alignment [1,2,3,4]. The precise attitude matrix is usually obtained with fine alignment stage. However, the recognized fine alignment methods, such as the Kalman-based alignment or gyrocompass alignment, would rely heavily on the coarse alignment accuracy. The higher coarse alignment accuracy can give the smaller initial system errors and provide a linearization condition, thereby guaranteeing the performance of fine alignment [5,6,7,8]. On the other hand, the in-motion coarse alignment (IMCA) aided by body-frame velocity has also drawn increasing attention [9,10,11,12]. Since the IMCA is an advantageous functionality for some urgent missions, such as military applications and natural disaster rescue, and so on. And the external ground velocity information relative to the body frame ($V^{b}$) is also easily obtained from other aiding sensors, e.g., Doppler velocity logs (DVL), odometers (OD), Beidou (BD) and GPS, etc.

For IMCA, the traditional analytic methods would be difficult to provide a roughly known initial attitude to the subsequent fine alignment because of the external disturbance and the vehicle maneuverability [13,14,15]. As a result, the in-motion attitude determination alignment (IMADA) has been presented to solve this problem [16,17]. With the attitude matrix decomposition technique, the vehicle maneuverability can be isolated. Meanwhile, the SINS attitude alignment problem is also equivalently transformed into the solution of constant initial attitude matrix. With the vector observations, the constant attitude matrix can be determined by the recursive Davenport’s q-method. Then, the problem of in-motion coarse alignment for body-frame velocity-aided SINS can be solved. And the IMADA method has also been proven to be the robustness and the anti-interference for the external disturbance [18,19]. 

Nevertheless, there are still some challenging issues for the body-frame velocity-aided IMADA, e.g., the slow convergence, the calculation errors, the vulnerability to divergence and the principled model errors [20,21,22]. Since the ground velocity information relative to the navigation frame ($V^{n}$) is only obtained indirectly with the inaccurate attitude matrix and the velocity information $V^{b}$, the convergence speed of the heading alignment would be slower inevitably as compared with the $V^{n}$-aided IMADA. Meanwhile the inaccurate attitude matrix would also result in the calculation errors, and further influence the alignment accuracy. Moreover, the noise disturbance of $V^{b}$ from the aiding sensors, such as DVL or OD, would also lead to the risk of divergence. Finally, the principled model errors are mainly caused by the omitted item ($\omega_{ie}^{b}\times V^{b}$) of alignment modeling. And the influence of the omitted item on alignment performance would increase with the vehicle velocity. As a result, the alignment performance of the $V^{b}$-aided IMADA may be degraded to a certain extent, thereby influencing the subsequent fine alignment.

For these aforementioned drawbacks of the $V^{b}$-aided IMADA, many works have been carried out. In [23,24], a fast IMADA assisted by the backward navigation algorithm is presented, thereby lengthening the SINS data equivalently and shortening the alignment time. Moreover, the low-pass finite impulse response (FIR) or infinite impulse response (IIR) filters have also been proposed to eliminate the noise disturbance of sensors, including the accelerometer sensor and the external aiding sensors [25,26,27]. In [28,29], the horizontal alignment algorithms based on the Gauss–Hermite filter or the gyrocompass alignment algorithm is applied to attenuate the noise disturbance of accelerometer and DVL, respectively. In [30], the attitude estimation with the Kalman filter is employed to track the attitude errors, thereby decreasing the calculation errors of IMADA. With the combination of two different velocity-based IMADAs (namely, $V^{b}$-based IMADA and $V^{n}$-based IMADA) and the multiple repeated alignment process, furthermore, the multistage IMADA (MIMADA ) has also been proposed to remove both the calculation errors and principal model errors and has superior performance [31]. In the implement process of MIMADA, however, the final alignment performance of $V^{b}$-aided IMADA would depend on the first level alignment performance significantly. Unluckily, the first level alignment accuracy is also difficult to predict and control. As a result, the final alignment performance of $V^{b}$-aided IMADA might not be improved, even deteriorated seriously. Further, the MIMADA proposed in [31] might be less meaning for practical engineering application.

Aiming at the above problem, this paper proposed an improved dual-velocity modeling-based MIMADA. With the designed dual velocity-modeling IMADA, the dependence degree of second-level alignment on the first level alignment performance can be weakened greatly. Further, the degradation phenomenon of the MIMADA can be reduced largely. As a result, a better statistic characteristic of alignment results can be obtained to improve the accuracy of coarse alignment and guarantee the reliability of alignment system. The remainder of this paper is organized as follows. In Section 2, the inherent designed defections of the traditional $V^{b}$-based IMADA are first analyzed. And the influence of the principled model errors on the alignment accuracy are verified with the Monte Carlo alignment simulation experiments. Then, a brief overview of the traditional MIMADA is presented. Subsequently, the existing problems of traditional MIMADA and the degradation phenomenon of alignment accuracy are also discussed and illustrated with the 30 groups of car-mounted alignment experiments. Section 3 is devoted to design the improved MIMADA with the proposed dual velocity-modeling IMADA. Experiments and simulations with the proposed MIMADA are carried out in Section 4, and the Section 5 is the conclusions.

## 2. Problem Statement

### 2.1. The Traditional Body-Frame Velocity-Aided In-Motion Attitude Determination Alignment

In this paper, we denote the initial frame by i, the Earth frame by e, the navigation frame by n, the body frame by b. Furthermore, the non-rotating inertial frames are denoted by n(0) and b(0), which are identical to the navigation frame and the body frame at time 0 respectively. The time-varying navigation frame and the time-varying body frame are denoted by n(t) and b(t), with time t. With the attitude matrix decomposition technique, the attitude matrix $C_{b}^{n}(t)$ can be written by [30]:

(1) $$C_{b}^{n}(t)=C_{b(t)}^{n(t)}={\left(C_{n(t)}^{n(0)}\right)}^{T}C_{b}^{n}(0)C_{b(t)}^{b(0)}$$

where the $C_{b}^{n}(0)$ is the initial attitude matrix between n-frame and b-frame at time 0, and it is also a constant matrix. Moreover, the other two matrixes $C_{n(t)}^{n(0)}$ and $C_{b(t)}^{b(0)}$ can also be updated in real time as follow [32]:

(2) $${\dot{C}}_{b(t)}^{b(0)}=C_{b(t)}^{b(0)}\left(\omega_{ib}^{b}\times \right)$$

(3) $${\dot{C}}_{n(t)}^{n(0)}=C_{n(t)}^{n(0)}\left(\omega_{in}^{n}\times \right)$$

where the initialization of $C_{n(t)}^{n(0)}(0)$ and $C_{b(t)}^{b(0)}(0)$ are all set to the identity matrix; $\omega_{ib}^{b}$ is gyroscopes outputs from the inertial measurement unit (IMU); $\omega_{in}^{n}=\omega_{ie}^{n}+\omega_{en}^{n}$. Since the coarse alignment is usually achieved with a short period of time, the change of vehicle position can be neglected and we have $L\approx L_{0}$. Then, the $\omega_{ie}^{n}$ and $\omega_{en}^{n}$ can be calculated by

(4) $$\left\{ \begin{array}{l} \omega_{ie}^{n}={\left[\begin{array}{ccc} 0 & \Omega \cos L_{0} & \Omega \sin L_{0} \end{array}\right]}^{T}\\ \omega_{en}^{n}={\left[\begin{array}{ccc} -V_{N}/R & -V_{E}/R & -V_{E}\tan L_{0}/R \end{array}\right]}^{T} \end{array}\right.$$

where $L_{0}$ is the initial latitude of vehicle; Ω denotes the angular rate of Earth’s rotation; R denotes the Earth radius; $V_{E}$ and $V_{N}$ are the East velocity and North velocity, respectively. Moreover, the vehicle velocity expressed in navigation frame $V^{n}$ is usually calculated by

(5) $$V^{n}={\hat{C}}_{b}^{n}(t)V^{b}$$

where ${\hat{C}}_{b}^{n}(t)$ is the calculated value of strapdown attitude matrix, and is usually acquired in the process of the coarse alignment. 

According to the analysis above, the SINS attitude alignment is also transformed into the solution of constant initial attitude matrix $C_{b}^{n}(0)$. With the traditional $V^{b}$-based IMADA, then, $C_{b}^{n}(0)$ can be determined by calculating the solution of the following observation equation [33]:

(6) $$\left\{ \begin{array}{l} C_{b}^{n}(0)\alpha_{v}^{b}=\beta_{v}^{b}\\ \alpha_{v}^{b}=C_{b(t)}^{b(0)}V^{b}(t)-V^{b}(0)+\int_{0}^{t}C_{b(t)}^{b(0)}\left(\omega_{ie}^{b}\times V^{b}\right)dt-\int_{0}^{t}C_{b(t)}^{b(0)}f^{b}dt\\ \beta_{v}^{b}=\int_{0}^{t}C_{n(t)}^{n(0)}g^{n}dt \end{array}\right.$$

where $f^{b}$ is the accelerometer outputs; $g^{n}={\left[\begin{array}{ccc} 0 & 0 & -g \end{array}\right]}^{T}$, g is the local gravity. For the solution of the observation equation, which is the known Wahba’s problem, the recursive Davenport’s q-method is applied here. As a result, the quaternion representation of the constant matrix $C_{b}^{n}(0)$ would be the largest positive eigenvalue of the matrix $K_{M}$ as follow:

(7) $$K_{M}=\left[\begin{array}{cc} B+B^{T}-\mathrm{tr}(B)I_{3} & \sum_{i=0}^{M}\beta_{i}\times \alpha_{i}\\ {\left(\sum_{i=0}^{M}\beta_{i}\times \alpha_{i}\right)}^{T} & \mathrm{tr}(B) \end{array}\right]$$

(8) $$B=\sum_{i=0}^{M}\beta_{i}\;\;\alpha_{i}^{T}$$

With the above description, the $V^{b}$-aided IMADA can be implemented. Since the $\omega_{ie}^{b}$ (the angular rate of Earth’s rotation expressed in b-frame) is difficult to obtain, however, the item $\omega_{ie}^{b}\times V^{b}$ of the observation vector $\alpha_{v}^{b}$ in Equation (6) is usually negligible. Consequently, the $\alpha_{v}^{b}$ is calculated approximatively as follows:

(9) $$\begin{array}{ll} \alpha_{v}^{b} & =C_{b(t)}^{b(0)}V^{b}(t)-V^{b}(0)+\int_{0}^{t}C_{b(t)}^{b(0)}\left(\omega_{ie}^{b}\times V^{b}\right)dt-\int_{0}^{t}C_{b(t)}^{b(0)}f^{b}dt\\ & \approx C_{b(t)}^{b(0)}V^{b}(t)-V^{b}(0)-\int_{0}^{t}C_{b(t)}^{b(0)}f^{b}dt \end{array}$$

As a result, the principled model errors (namely, the approximationerror $\omega_{ie}^{b}\times V^{b}$) would occur in $V^{b}$-aided IMADA, thereby influencing the alignment accuracy. Moreover, it is easy to see that those influences are increasing with the vehicle velocity $V^{b}$. In the process of coarse alignment, on the other hand, the SINS attitude matrix usually would have the lower accuracy. Further, the process attitude matrix is employed to calculate navigation-frame velocity $V^{n}$ from Equation (5) and update the rotation matrix $C_{n(t)}^{n(0)}(0)$ from Equation (3), which would also result in the large calculation errors, thereby influencing the alignment performance [31].

**Figure 1:**
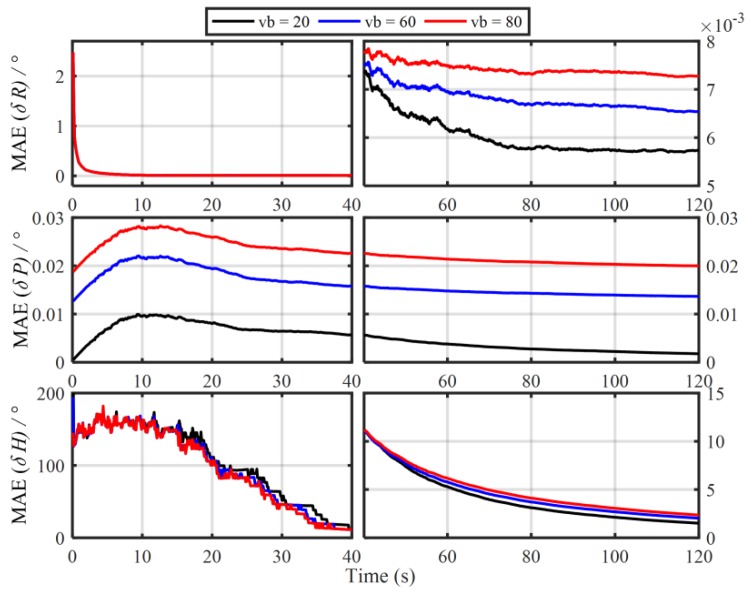
The mean absolute deviation (MAE) curves of 50 alignment results with the traditional $V^{b}$-based IMADA.

**Figure 2:**
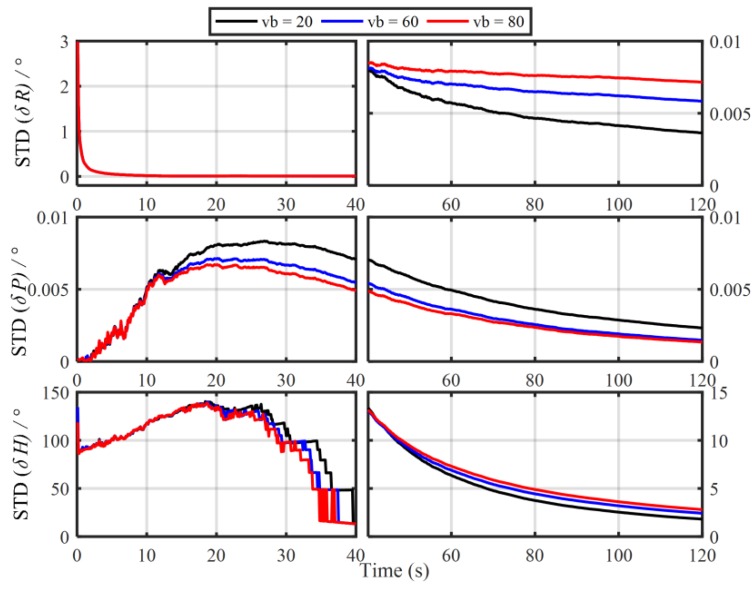
The standard deviation (STD) curves of 50 alignment results with the traditional $V^{b}$-based IMADA.

In order to verify the influence of the principled model errors of $V^{b}$-based IMADA on the alignment accuracy, the Monte Carlo simulation experiments with the different vehicle velocities are carried out here. Total of 50 groups of 120 s simulation data are generated respectively from the SINS simulator [31]. The main simulation parameters are shown in Table 1. The sample frequency of IMU is 100 Hz. The vehicle sails at the different speed values of 20 m/s, 60 m/s, 80 m/s, respectively, and the generated external aiding velocity information $V^{b}$ is added intentionally by the Gaussian white noise of standard deviation 0.03 m/s. With the above $V^{b}$-based IMADA, then, the curves of mean absolute deviation (MAE) and standard deviation (STD) of 50 alignment errors are shown in Figure 1 and Figure 2. The statistics of 50 heading alignment errors are also shown in Table 2.

From Figure 1 and Figure 2, the MAE and STD curves of three-axis alignment errors are all convergent with the time. As a result, the $V^{b}$-aided IMADA can solve the in-motion coarse alignment problem. However, it is also easy to see that the alignment accuracy would degrade with the vehicle velocity. The MAE and STD of 50 alignment results with the different speeds of 20 m/s, 60 m/s, 80 m/s are 1.5125°, 2.0287, 2.3574 and 1.8170°, 2.4259°, 2.8069°, respectively. From Table 2, on the other hand, the statistic characteristic also becomes worse with the velocity. The maximum of absolute value of 50 heading alignment errors are 4.0657°, 5.5640°, 6.3852°, respectively. This is coincident with the above analysis about the $V^{b}$-based IMADA. Because of the omitted item $\omega_{ie}^{b}\times V^{b}$, the presented principled model errors would influence the alignment accuracy, and this degradation aggravates with the vehicle speed. Then, the worse statistic characteristic would result in a lower reliability for SINS alignment system. This would be disadvantageous for the practical engineering applications. As a result, the principled model errors arising in the traditional $V^{b}$-based IMADA must be eliminated, especially for high-speed vehicle.

### 2.2. The Ttraditional MultistageIn-Motion Attitude Determination Alignment and the Aegradation Phenomenon

As mentioned and demonstrated above, the principled model errors and the calculation errors of the traditional $V^{b}$-aided IMADA would degrade the alignment accuracy and influence the reliability of the initial alignment system. As a result, those two error sources should be removed to improve the performance of the initial alignment system. In [31], then, the $V^{n}$-aided IMADA without the principled model errors is employed tactfully to solve the inherent designed defections of the $V^{b}$-based IMADA. The multistage in-motion attitude determination alignment (MIMADA) with two different velocity models is presented. By integrating the $V^{b}$-based IMADA and the $V^{n}$-based IMADA, as well as the multiple repeated alignment process, the MIMADA can eliminate effectively the principled model errors and the calculation errors, thereby improving the alignment performance. 

To be more specific, the traditional $V^{b}$-based IMADA is first carried out to acquire a final value of initial constant attitude matrix $C_{b}^{n}{\left(0\right)}_{1}$, which would be a higher accuracy value. In the second-level alignment process, then, the first final value $C_{b}^{n}{\left(0\right)}_{1}$ is utilized to calculate the stapdown attitude matrix $C_{b}^{n}(t)$ by updating Equation (1). Hence, the calculated errors of stapdown attitude matrix can be decreased as compared with the traditional method with the process value of the constant attitude matrix $C_{b(t)}^{n(t)}(0)$, which is updated recursively with the IMADA and has worse accuracy. As a result, the influence of the calculation errors on the $V^{b}$-aided IMADA can also be decreased, thereby improving the alignment performance. Further, the real-time navigation-frame velocity $V^{n}$ can also be obtained with the body-frame velocity $V^{b}$ from Equation (5) and have higher calculation accuracy. Meanwhile, the $V^{n}$-based IMADA can also be implemented and can be applied to avoid the principled model errors of traditional $V^{b}$-based IMADA. With the latest final value of higher accuracy constant attitude matrix, moreover, the $V^{n}$-based IMADA is conducted repeatedly to further decrease and gradually remove the calculated errors. The more details about MIMADA can be found in [31]. According to the above description, the MIMADA can eliminate the principled model errors and the calculation errors of the traditional $V^{b}$-aided IMADA and has superior performance. 

For the $V^{n}$-based IMADA, on the other hand, the observation equation of attitude determination is also presented as follows [13]:

(10) $$\left\{ \begin{array}{l} C_{b}^{n}(0)\alpha_{v}^{n}=\beta_{v}^{n}\\ \alpha_{v}^{n}=\int_{0}^{t}C_{b(t)}^{b(0)}f_{ib}^{b}dt\\ \beta_{v}^{n}=C_{n(t)}^{n(0)}V^{n}-V^{n}(0)+\int_{0}^{t}C_{n(t)}^{n(0)}\omega_{ie}^{n}\times V^{n}dt-\int_{0}^{t}C_{n(t)}^{n(0)}g^{n}dt \end{array}\right.$$

From Equation (10), the in-motion coarse alignment with the $V^{n}$-based IMADA would be implemented and have no principled errors. Then, the MIMADA proposed in [31] can also be implemented to solve the existing inherent problem of the traditional $V^{b}$-based IMADA, thereby improving the alignment performance. 

**Figure 3:**
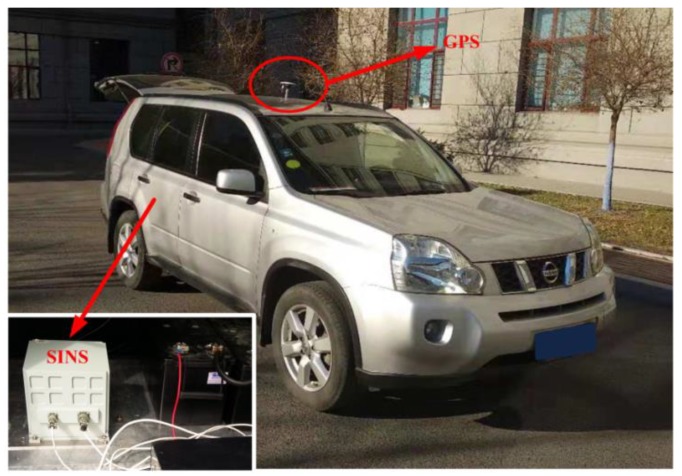
The car-mounted experiment and equipments.

**Figure 4:**
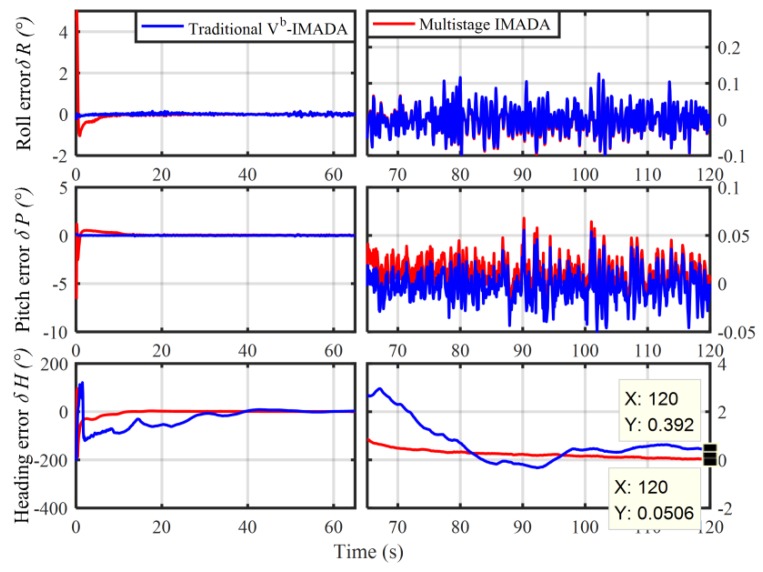
The improvement of alignment accuracy for the traditional multistage IMADA (MIMADA).

In order to verify the superior performance of MIMADA, the car-mounted experiment was carried out. The experimental equipments and the main parameters are shown in Figure 3 and Table 3, respectively. Here, the integrated navigation system with both the differential GPS and the navigation-grade fiber optic gyroscope (FOG) SINS served as the attitude benchmark for the in-motion coarse alignment and provided the external aided velocity information $V^{b}$. A total of 120 s coarse alignment test is executed and operated in Harbin (126° E,46° N), Heilongjiang Province, China. Here, only the second-level alignment for MIMADA is carried out. If not explicitly stated, the follow MIMADA experiments are all achieved by the second-level alignment. With the traditional $V^{b}$-aided IMADA and the multistage IMADA, the three-axis alignment errors are shown in Figure 4. 

In Figure 4, the three-axis alignment errors are all convergence with time. As a result, the MIMADA proposed in [31] can be applied to achieve the in-motion alignment for body-frame velocity-aided SINS. Moreover, the alignment errors (roll, pitch, and heading) in 120 s with both the IMADA and MIMADA algorithms are −0.0262°,−0.0062°,0.3920°, and −0.0278°,0.0032°,0.0506°, respectively. Hence, the MIMADA can improve the alignment accuracy. This is because that the MIMADA removes the principled model errors and the calculation errors, thereby eliminating the existing inherent defections of the $V^{b}$-based IMADA and improving the alignment performance. Therefore, the MIMADA would have superior performance as described above.

**Figure 5:**
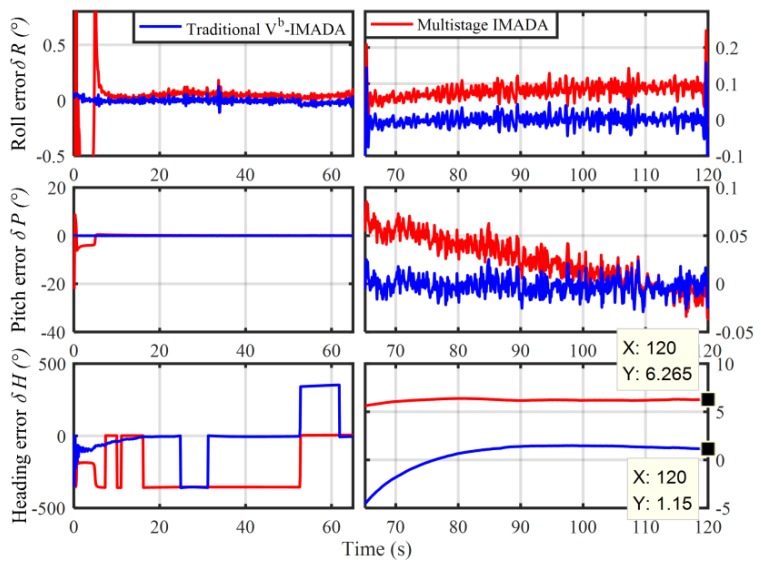
The degradation phenomenon of alignment accuracy for the traditional MIMADA.

Nonetheless, it should be still remarkable that the performance of the second-level and the higher level alignments with MIMADA would depend on the first-level alignment performance significantly. Since the performance of the $V^{n}$-based IMADA would mainly be influenced by the accuracy of the navigation-frame velocity $V^{n}$ according to Equation (10). In MIMADA, however, $V^{n}$ would only be obtained by the first final value of initial constant attitude matrix $C_{b}^{n}{\left(0\right)}_{1}$ and the body-frame velocity $V^{b}$. If the first-level alignment performance is worse, therefore, the degradation of the alignment performance would possibly arise in MIMADA to a certain extent. With the same experimental equipments above, for example, another 120 s coarse alignment test is also conducted. A degradation phenomenon would occur and the alignment results are shown in Figure 5. 

From Figure 5, it is obvious that the alignment accuracy would be worse, not improving, when the MIMADA is applied to achieve coarse alignment. The heading alignment errors with two algorithms in 120 s are 1.15° and 6.265°, respectively. As a result, it is contrary to the designed expectation of the MIMADA. Further, the MIMADA proposed in [31] might be less appropriate for practical engineering application.

**Figure 6:**
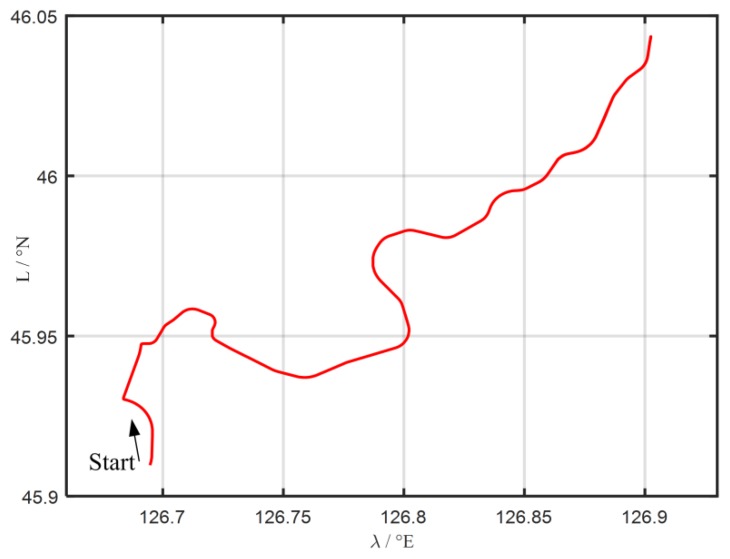
The test trajectory of vehicle experiment.

**Figure 7:**
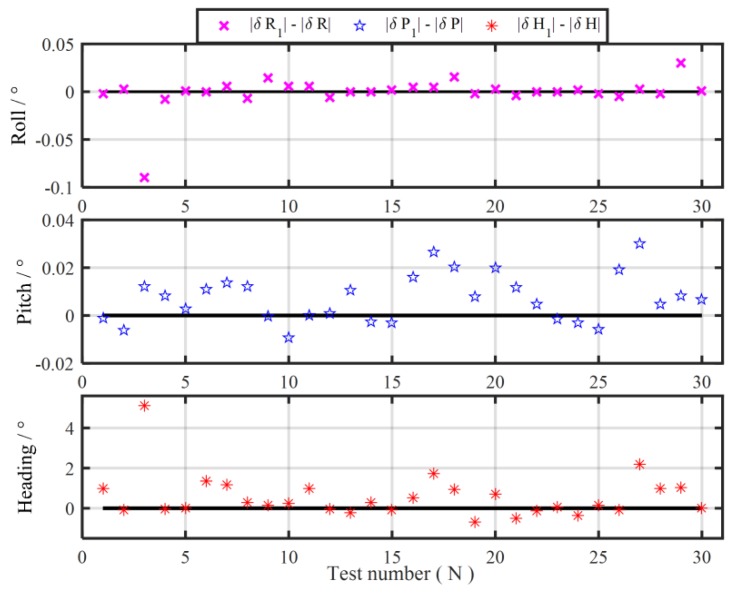
The difference of absolute value of alignment errors between the MIMADA and the $V^{b}$-aided IMADA.

Without loss of generality, other 30 groups 120 s coarse alignment tests are also carried out to further illustrate the degradation phenomenon of the traditional MIMADA. With same experimental equipments above, the total 60-min test data are collected. The test trajectory is shown in Figure 6. Then, those test data are divided successively into 30 groups 120 s data segments. The in-motion coarse alignments with the two algorithms are all conducted. The 30 differences of absolute value of alignment errors between the MIMADA and traditional $V^{b}$-aided IMADA in 120 s are all shown in Figure 7. The subscript 1 denotes alignment results with the MIMADA. In Figure 7, obviously, the difference values greater than 0 mean that the alignment results are degraded with MIMADA. Whereas, the difference values less than 0 mean that the alignment results are improved. Moreover, the statistics of the 30 alignment results are also shown in Table 4.

From Figure 7, it is easy to see that the degradation number for alignment performance with MIMADA would be more than the improvement number. From Table 4, the degradation number of heading alignment would be 20 in 30 alignment experiments. Even the maximum value of degradation of alignment accuracy is 5.1148°. Then the performance of the subsequent fine alignment might be influenced seriously because of the poor coarse alignment accuracy. Moreover, the mean and variance of the differences of absolute value of heading alignment errors are 0.5523° and 1.0928°. As a result, the statistic characteristics of alignment accuracy with MIMADA would be bad, thereby decreasing the reliability of alignment system. 

This is because that the performance of MIMADA would mainly depend on the first level alignment performance as analyzed above, thereby resulting in the degradation phenomenon. Thus, the final alignment accuracy would be influenced. As a result, the first level alignment accuracy must be guaranteed strictly. Otherwise, the MIMADA might be less appropriate for practical application because of the degradation phenomenon. Because of unavoidable random noises of the sensors, however, the first-level alignment accuracy with the traditional $V^{b}$-aided IMADA is usually difficult to guarantee and control. Moreover, the higher requirement for the first-level alignment accuracy may cost more alignment time, which is contradiction to the rapidness of the initial alignment. Therefore, a new solution for MIMADA is expected to be proposed to guarantee the statistic characteristics. 

## 3. The Improved Multistage In-Motion Attitude Determination Alignment with the Dual Velocity-Modeling

In this section, an improved MIMADA is presented to solve the aforementioned drawbacks of traditional MIMADA. The designed dual velocity-modeling IMADA is proposed to substitute the $V^{n}$-based IMADA in the process of the high-level alignment for traditional MIMADA. The degradation phenomenon is expected to be decreased. The desired statistic characteristics can also be guaranteed. The detail algebraic expressions for dual velocity-modeling IMADA are derived as follows:

Differentiating both sides of Equation (5), we have:

(11) $${\dot{V}}^{n}=C_{b}^{n}{\dot{V}}^{b}+{\dot{C}}_{b}^{n}V^{b}$$

Then, substituting the formula ${\dot{C}}_{b}^{n}=C_{b}^{n}(\omega_{nb}^{b}\times )$ into the Equation (11) gives,

(12) $${\dot{V}}^{n}=C_{b}^{n}({\dot{V}}^{b}+\omega_{nb}^{b}\times V^{b})$$

Moreover, taking the cross-product in Equation (5), we have

(13) $$(\omega_{ie}^{n}+\omega_{in}^{n})\times V^{n}=C_{b}^{n}\left[(\omega_{ie}^{b}+\omega_{in}^{b})\times V^{b}\right]$$

On the other hand, the force equation for SINS is well-known as

(14) $${\dot{V}}^{n}=C_{b}^{n}f_{ib}^{b}-(2\omega_{ie}^{n}+\omega_{en}^{n})\times V^{n}+g^{n}$$

Then, substituting Equations (12) and (13) into the force equation and reorganizing, we have

(15) $$C_{b}^{n}\left[{\dot{V}}^{b}+\omega_{ib}^{b}\times V^{b}-f_{ib}^{b}\right]=g^{n}-\omega_{ie}^{n}\times V^{n}$$

Substituting Equation (1) into Equation (15), multiplying the left by $C_{n(t)}^{n(0)}$ and integrating both sides, we have

(16) $$C_{b}^{n}(0)\int_{0}^{t}C_{b(t)}^{b(0)}\left({\dot{V}}^{b}+\omega_{ib}^{b}\times V^{b}-f_{ib}^{b}\right)dt=\int_{0}^{t}C_{n(t)}^{n(0)}\left(g^{n}-\omega_{ie}^{n}\times V^{n}\right)dt$$

Since

(17) $$\begin{array}{cc} \int_{0}^{t}C_{b(t)}^{b(0)}{\dot{V}}^{b}dt= & C_{b(t)}^{b(0)}{V^{b}|}_{0}^{t}-\int_{0}^{t}C_{b(t)}^{b(0)}\omega_{ib}^{b}\times V^{b}dt\\ & =C_{b(t)}^{b(0)}V^{b}(t)-V^{b}(0)-\int_{0}^{t}C_{b(t)}^{b(0)}\omega_{ib}^{b}\times V^{b}dt \end{array}$$

The Equation (17) can be rewritten as,

(18) $$C_{b}^{n}(0)\left[C_{b(t)}^{b(0)}V^{b}(t)-V^{b}(0)-\int_{0}^{t}C_{b(t)}^{b(0)}f_{ib}^{b}dt\right]=\int_{0}^{t}C_{n(t)}^{n(0)}\left(g^{n}-\omega_{ie}^{n}\times V^{n}\right)dt$$

According to Equation (18), further, the observation equation of attitude determination with the dual velocity-modeling can be constructed as follows:

(19) $$\left\{ \begin{array}{l} C_{b}^{n}(0)\alpha_{v}^{bn}=\beta_{v}^{bn}\\ \alpha_{v}^{bn}=C_{b(t)}^{b(0)}V^{b}(t)-V^{b}(0)-\int_{0}^{t}C_{b(t)}^{b(0)}f_{ib}^{b}dt\\ \beta_{v}^{bn}=\int_{0}^{t}C_{n(t)}^{n(0)}\left(g^{n}-\omega_{ie}^{n}\times V^{n}\right)dt \end{array}\right.$$

With the above designed dual velocity-modeling IMADA, the initial constant matrix $C_{b}^{n}(0)$ can be determined by the recursive Davenport’s q-method.

Comparing with Equations (9) and (19), however, obviously the omitted item of alignment modeling would never have been developed in the dual velocity-modeling IMADA. With the proposed IMADA, consequently, the principled model errors for the traditional $V^{b}$-based IMADA would also be avoided, thereby improving the coarse alignment accuracy and providing a better initial condition for the subsequent fine alignment. From Equation (19), moreover, only the item $\omega_{ie}^{n}\times V^{n}$ is related to the navigation-frame velocity $V^{n}$ in the proposed dual velocity-modeling IMADA. Hence the dependence degree of the alignment performance on the ground velocity $V^{n}$ can be decreased largely as compared with the $V^{n}$-based IMADA in Equation (10). For the MIMADA aided by body-frame velocity $V^{b}$, further, the ground velocity $V^{n}$ is usually obtained with the first final value of initial constant attitude matrix $C_{b}^{n}{\left(0\right)}_{1}$ and the body-frame velocity $V^{b}$. Hence, the accuracy requirement for the first-level alignment in MIMADA can be weakened greatly accordingly when the dual velocity-modeling IMADA are applied to substitute the $V^{n}$-based IMADA in the process of the high-level alignment for the MIMADA. Furthermore, the Earth angular rate $\omega_{ie}^{n}$ is also relatively smaller. Even if there is still deterioration, therefore, the accuracy degradation of the proposed dual velocity-modeling IMADA would be less, which is mainly caused by the addition item $\omega_{ie}^{n}\times V^{n}$. Consequently, the dual velocity-modeling IMADA can be employed to substitute the $V^{n}$-based IMADA for the high-level alignment and to solve the practical application problem for traditional MIMADA as mentioned and demonstrated above.

**Figure 8:**
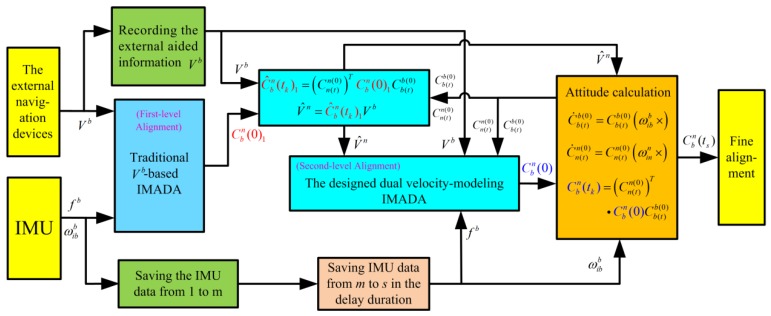
The block diagram of the improved multistage IMADA with the designed dual velocity-modeling IMADA.

On the other hand, the block diagram of the improved multistage IMADA based on the designed dual velocity-modeling IMADA with the second levels is also illustrated in Figure 8. As shown in Figure 8, the initial constant attitude matrix $C_{b}^{n}{\left(0\right)}_{1}$ can be first obtained from the first-level alignment with the traditional $V^{b}$-based IMADA. Then, the higher accuracy matrix $C_{b}^{n}{\left(0\right)}_{1}$ can be applied to acquire the navigation-frame velocity ($V^{n}$) with the less calculation errors. Subsequently, the matrix $C_{n(t)}^{n(0)}(t)$ can also be updated accurately. The designed dual velocity-modeling IMADA can also be implemented to accomplish the second-level alignment. Further, the alignment process is carried out from 1 to s to obtain initial strapdown matrix $C_{b}^{n}(t_{s})$. Then the SINS starts the fine alignment process. 

According to the above analysis, therefore, the proposed dual velocity-modeling IMADA can be applied to implement the MIMADA and have less degradation phenomenon. The MIMADA based on the designed dual velocity-modeling IMADA would be promising for achieving the in-motion coarse alignment without sacrificing the statistic characteristics for body-frame velocity-aided SINS, thereby improving the alignment performance of traditional MIMADA and guaranteeing the reliability of alignment system. 

## 4. Experiments and Simulations

**Figure 9:**
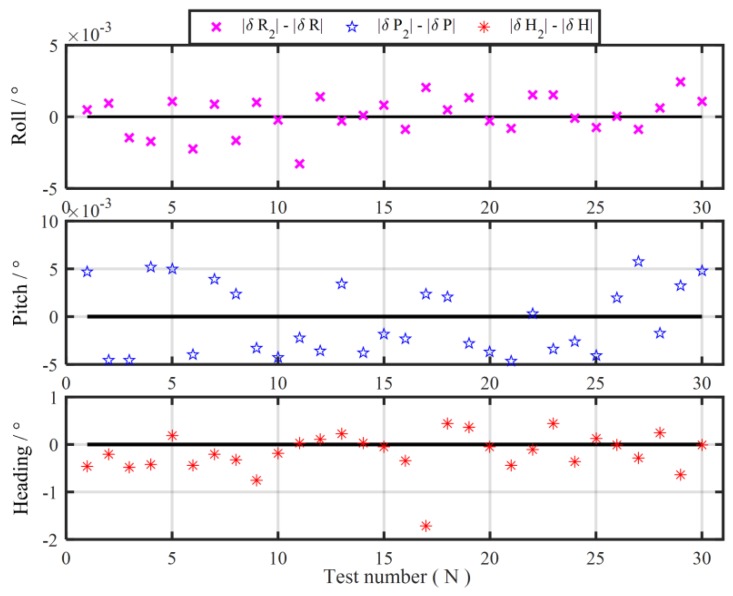
The difference of absolute value of alignment errors between the proposed MIMADA and the $V^{b}$-aided IMADA.

**Figure 10:**
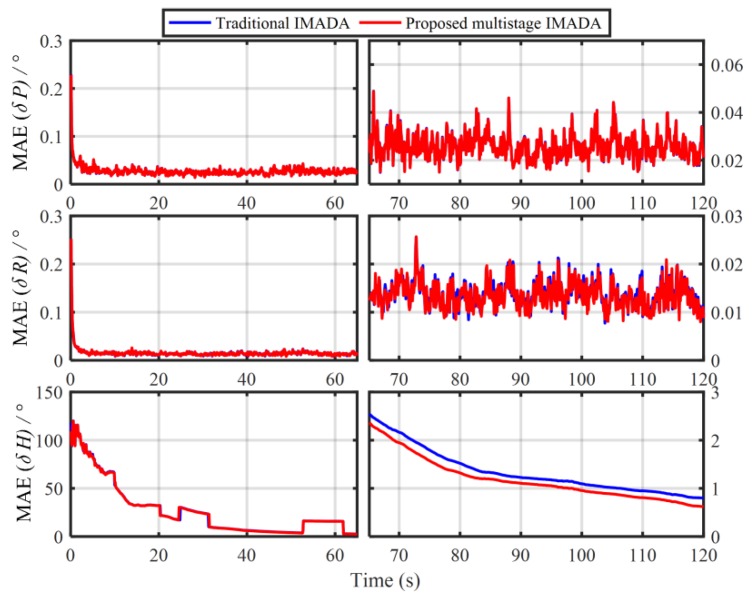
The MAE comparison of 30 alignment experiments between the proposed MIMADA and the traditional $V^{b}$-aided IMADA.

**Figure 11:**
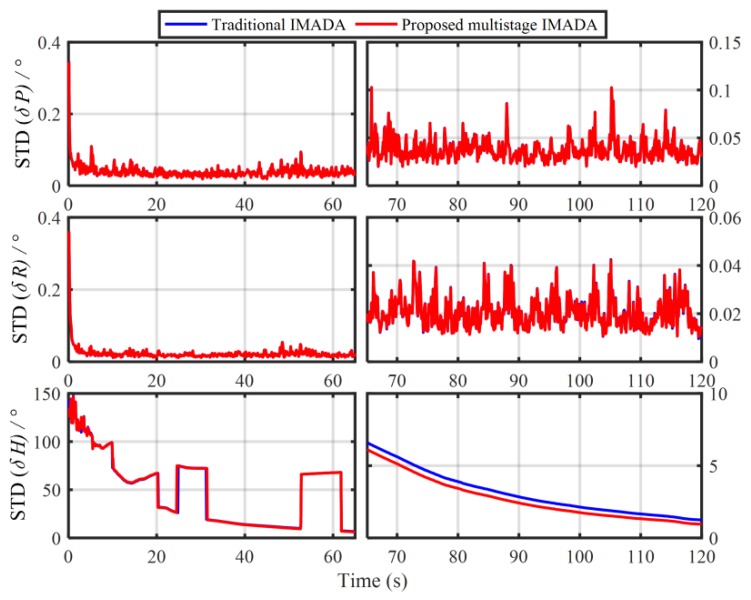
The STD comparison of 30 alignment experiments between the proposed MIMADA and the traditional $V^{b}$-aided IMADA.

In order to demonstrate the validity of the improved MIMADA based on the dual velocity-modeling IMADA, the car-mounted experiments and simulations are conducted in this section. With the same test data in Section 2.2, the 30 group in-motion coarse alignment experiments are also performed similarly to illustrate the weak degradation phenomenon and the superior performance of the proposed MIMADA. The 30 differences of absolute value of alignment errors between the improved MIMADA and traditional $V^{b}$-based IMADA in 120 s are shown in Figure 9. The subscript 2 denotes alignment results with the proposed MIMADA. Similarly, the difference values greater than 0 mean the degradation, whereas, the difference values less than 0 mean the improvement. Moreover, the statistics of the 30 alignment results are shown in Table 5. The curves of the MAE and the STD of 30 alignment results with the improved MIMADA and traditional $V^{b}$-based IMADA are also shown in Figure 10 and Figure 11. 

Comparing Figure 7 and Figure 9, it is easy to see that the numbers of the difference values greater than 0 is obviously less than the ones with the traditional MIMADA. As a result, the degradation phenomenon with the proposed MIMADA can be decreased largely. In the total 30 alignment results, the degradation numbers of heading alignment with two MIMADA algorithms are 10 and 20, respectively. The maximum degradation of heading error with the proposed MIMADA is only 0.4431°, which is also a very small value, while the maximum degradation of heading accuracy with the traditional algorithm is 5.1148° from Table 4. Therefore, the designed dual velocity-modeling IMADA can decrease the dependence degree on the accuracy requirement of the first-level alignment, which can be expected to improve the alignment performance of traditional $V^{b}$-based IMADA without sacrificing the statistic characteristics. This coincides with the previous analysis for the improved MIMADA.

From Table 5, on the other hand, the mean of the differences of absolute value of heading alignment errors is a negative value and is −0.1767°, while the mean of the differences with the traditional MIMADA is 0.5523° and is a positive number, as shown in Table 4. As a result, the proposed MIMADA can solve the drawbacks of traditional MIMADA and decrease the degradation phenomenon, thereby guaranteeing the reliability of alignment system. The proposed MIMADA can also improve the traditional $V^{b}$-based IMADA and has a better statistical performance. Consequently, the improved MIMADA would have higher meaning for practical application and result in the superior performance.

From Figure 10 and Figure 11, we can see that the MAE and STD curves of alignment errors with the traditional $V^{b}$-based IMADA and the proposed MIMADA show convergence with time. As a result, the proposed MIMADA can achieve the in-motion coarse alignment for body-frame velocity-aided SINS. Nonetheless, the MAE and STD with the proposed MIMADA is also obviously less than the ones with the $V^{b}$-based IMADA. The MAEs and STDs of 30 heading alignment errors with two algorithms in 120 s are 0.6174°, 0.9483° and 0.7991°, 1.2435°. This coincides with the previous analysis for the superior performance of the improved MIMADA. Since the proposed MIMADA can remove the principled model errors and the calculation errors of the traditional $V^{b}$-based IMADA, thereby improving the alignment accuracy. Meanwhile, the dual velocity-modeling IMADA can reduce the dependence degree on the accuracy requirement of the first-level alignment, thereby decreasing the degradation phenomenon of the traditional MIMADA. As a result, the improved MIMADA not only can solve the inherent defections of the $V^{b}$-based IMADA, but also have a better statistic characteristics, thereby resulting in the superior performance.

**Figure 12:**
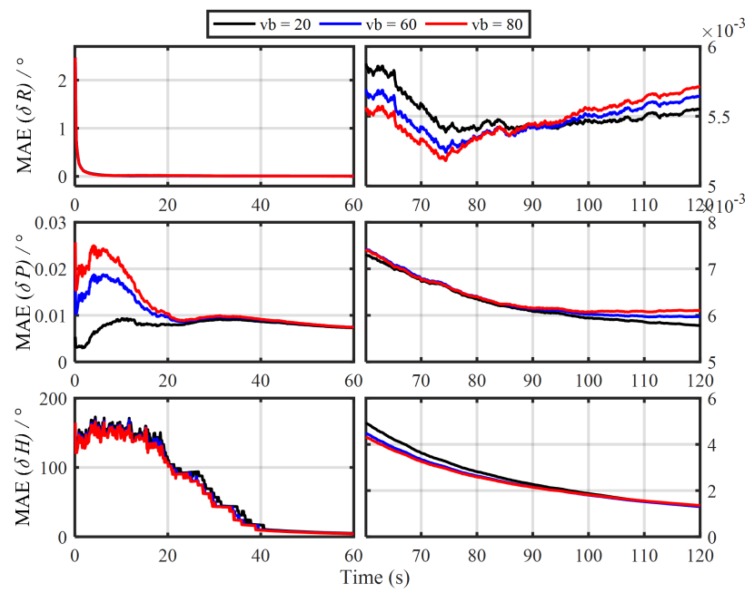
The MAE curves of 50 alignment results with the proposed MIMADA.

**Figure 13:**
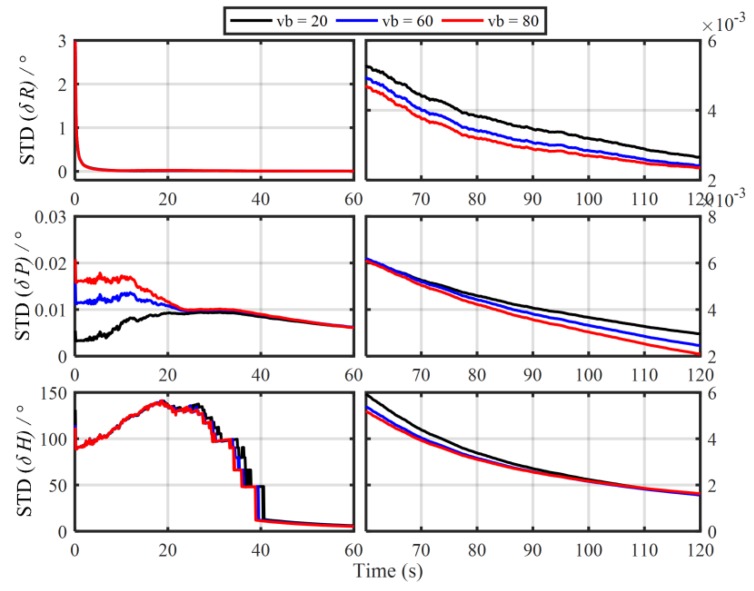
The STD curves of 50 alignment results with the proposed MIMADA.

In order to verify the superior performance of the proposed MIMADA further, moreover, the in-motion coarse alignment simulation experiments with the different vehicle velocities are also carried out. With the same simulation data in Section 2.1, the 50 Monte Carlo initial alignment simulations for body-frame velocity-aided SINS are also presented by using the proposed MIMADA. Then, the MAE and STD curves of 50 alignment results are shown in Figure 12 and Figure 13. Moreover, the statistics of 50 heading alignment errors in 120 s are also shown in Table 6.

From Figure 12 and Figure 13, the alignment errors with the different vehicle velocities, all show convergence with time. Comparing Figure 1 and Figure 12 and Figure 2 and Figure 13 respectively, even for the different vehicle-velocity in-motion initial alignments, the final alignment errors with the proposed MIMADA in 120 s are still almost the same. The heading alignment accuracy with the different speeds of 20 m/s, 60 m/s, 80 m/s are 1.3063 °, 1.3102°, 1.3564°, respectively. As a result, the proposed MIMADA can solve the existing problem of traditional $V^{b}$-based IMADA, where the alignment accuracy would degrade with the vehicle velocity. Comparing Table 2 and Table 6, moreover, the statistics of the 50 alignment errors with the proposed MIMADA are also better than the ones with traditional $V^{b}$-based IMADA. The MAEs of the heading alignment accuracy with two MIMADA algorithms in 20 m/s are 1.3063° and 1.5125°, respectively. This coincides with the previous analysis. With the proposed MIMADA, the principled model errors and the calculation errors of the traditional $V^{b}$-based IMADA can be removing, thereby improving the alignment accuracy. With the designed dual velocity-modeling IMADA, meanwhile, the degradation phenomenon of the traditional MIMADA can also be decreased largely, thereby guaranteeing the better statistic characteristics and resulting in the reliability of alignment system further. As a result, the proposed MIMADA would have the superior performance and the higher engineering value. 

## 5. Conclusions

The existing principled model errors and calculation errors for $V^{b}$-based IMADA would degrade the alignment accuracy inevitably, especially for high-speed in-motion alignment, thereby further influencing the performance of fine alignment. The newly-presented MIMADA can eliminate the inherent defections of traditional $V^{b}$-based IMADA and improve the alignment performance. However, the exhibited degradation phenomenon would result in the worse statistic characteristics for the finial alignment results, and hence decrease the reliability for alignment system. As a result, this paper proposes an improved multistage in-motion attitude determination alignment to achieve the IMCA for body-frame velocity-aided SINS, where the dual velocity-modeling IMADA is designed to execute the second-level alignment and the high-level alignment for traditional MIMADA. With the proposed dual velocity-modeling IMADA, the dependence degree of second-level alignment on the first level alignment performance can be weakened greatly as compared with the traditional multistage IMADA. Further, the degradation phenomenon of traditional MIMADA can also be reduced largely. Not only the designed drawbacks of traditional $V^{b}$-based IMADA can be solved, but also the statistic characteristics of alignment results can also been guaranteed. Moreover, the worse alignment accuracy for the $V^{b}$-based IMADA, the degradation phenomenon for the traditional MIMADA and the superior performance for the proposed MIMADA are demonstrated by the 30 groups of car-mounted experiments and the Monte Carlo simulation experiments. The results show that the number of the heading degradation of the second-level alignment is reduced to 10 as compared to the traditional number 20. Moreover, the alignment accuracy of heading is improved by 23%. Even with the different speeds of 20 m/s, 60 m/s, 80 m/s, the heading alignment accuracies are 1.3063 °, 1.3102°, 1.3564° and are still almost the same. Thus, the proposed multistage IMADA can improve the alignment accuracy without sacrificing the statistic characteristics, thereby guaranteeing the reliability of alignment system, superior performance, and higher engineering value.

## Figures and Tables

**Table 1:** The specifications of simulation parameters.

Parameters	Specifications
Initial position	Latitude: $L_{0}=45.6778{}^{\circ}$, Longitude: $\lambda_{0}=126.6778{}^{\circ}$
Initial attitude	Roll: 0°, Pitch: 0°, Heading: 30°
IMU	Gyroscope: constant drift 0.01°/h, random noise 0.001°/h
	Accelerometer: constant bias 100 μg, random noise 10 μg

**Table 2:** Statistics of the 50 heading alignment errors with the $V^{b}$-based in-motion attitude determination alignment (IMADA) in 120 s.

Heading Alignment Errors	MAE (°)	Mean (°)	STD (°)	Maximum (°)	Minimum (°)	Max of Absolute Value (°)
$V^{b}=20$ m/s	1.5125	0.0468	1.8170	2.8012	−4.0657	4.0657
$V^{b}=60$ m/s	2.0287	0.3347	2.4259	3.0202	−5.5640	5.5640
$V^{b}=80$ m/s	2.3574	0.4700	2.8069	3.2874	−6.3852	6.3852

**Table 3:** The specifications of car-mounted experimental parameters.

Parameters	Specifications
IMU	Gyroscope: constant drift less 0.01°/h, random noise less 0.001°/h
	Accelerometer: constant bias less 100 μg, random noise less 10 μg
Differential GPS	Velocity: 0.05 m/s (RMS)
	Position: 0.1 m (RMS)

**Table 4:** Statistics of 30 alignment results with MIMADA and traditional $V^{b}$-aided IMADA.

The Differences of Absolute Value of Alignment Errors	Mean (°)	Variance (°)	Maximum (°)	Minimum (°)	Number of Degradation (>0)
$\left|\delta R_{1}\right|-\left|\delta R\right|$	−0.0009	0.0183	0.0298	−0.0894	13
$\left|\delta P_{1}\right|-\left|\delta P\right|$	0.0071	0.0099	0.0301	−0.0091	19
$\left|\delta H_{1}\right|-\left|\delta H\right|$	0.5523	1.0928	5.1148	−0.6906	20

**Table 5:** Statistics of 30 alignment results with the proposed MIMADA and traditional $V^{b}$-aided IMADA.

Differences of Absolute Value of Alignment Errors	Mean (°)	STD (°)	Maximum (°)	Minimum (°)	Number of Degradation(>0)
$\left|\delta R_{2}\right|-\left|\delta R\right|$	0.0001	0.0013	0.0024	−0.0033	15
$\left|\delta P_{2}\right|-\left|\delta P\right|$	−0.0004	0.0037	0.0057	−0.0047	12
$\left|\delta H_{2}\right|-\left|\delta H\right|$	−0.1767	0.4261	0.4431	−1.7122	10

**Table 6:** Statistics of the 50 heading alignment errors with the proposed MIMADA in 120 s.

The Heading Alignment Errors	MAE (°)	Mean (°)	STD (°)	Maximum (°)	Minimum (°)	Max of Absolute Value (°)
$V^{b}=20$ m/s	1.3063	0.1030	1.5675	2.8094	−3.3243	3.3243
$V^{b}=60$ m/s	1.3102	0.0535	1.5715	2.6882	−3.3842	3.3842
$V^{b}=80$ m/s	1.3564	0.0113	1.6258	2.6716	−3.5307	3.5307
